# The potential of liposome–encapsulated ciprofloxacin as a tularemia therapy

**DOI:** 10.3389/fcimb.2014.00079

**Published:** 2014-06-18

**Authors:** Karleigh A. Hamblin, Jonathan P. Wong, James D. Blanchard, Helen S. Atkins

**Affiliations:** ^1^Microbiology Group, Defence Science and Technology Laboratory, Biomedical Sciences Department, Porton Down, Salisbury, UK; ^2^Defence Research and Development Canada, Suffield Research Center, Ralston, AL, Canada; ^3^Aradigm Corporation, Hayward, CA, USA

**Keywords:** tularemia, liposomal, ciprofloxacin, *Francisella tularensis*

## Abstract

Liposome-encapsulation has been suggested as method to improve the efficacy of ciprofloxacin against the intracellular pathogen, *Francisella tularensis*. Early work with a prototype formulation, evaluated for use against the *F. tularensis* live vaccine strain, showed that a single dose of liposomal ciprofloxacin given by the intranasal or inhalational route could provide protection in a mouse model of pneumonic tularemia. Liposomal ciprofloxacin offered better protection than ciprofloxacin given by the same routes. Liposomal ciprofloxacin has been further developed by Aradigm Corporation for *Pseudomonas aeruginosa* infections in patients with cystic fibrosis and non-cystic fibrosis bronchiectasis. This advanced development formulation is safe, effective and well tolerated in human clinical trials. Further evaluation of the advanced liposomal ciprofloxacin formulation against the highly virulent *F. tularensis* Schu S4 strain has shown that aerosolized CFI (Ciprofloxacin encapsulated in liposomes for inhalation) provides significantly better protection than oral ciprofloxacin. Thus, liposomal ciprofloxacin is a promising treatment for tularemia and further research with the aim of enabling licensure under the animal rule is warranted.

## Introduction

The extremely low infectious dose and severe disease following inhalation previously led to the development of *Francisella tularensis* as a biological weapon by Russia, Japan, and the USA (Dennis et al., 2001). To combat the threat of a deliberate release, there is a need for an easily administered therapy which can be used in the event of a mass casualty situation. The latest consensus statement on tularemia as a biological weapon suggested ciprofloxacin or doxycycline should be given in the event of a mass casualty situation (Dennis et al., 2001).

Ciprofloxacin, a broad spectrum second generation fluoroquinolone, has been used to treat infections for over 20 years. Tularemia outbreaks in Spain (Perez-Castrillon et al., 2001) and Turkey (Meric et al., 2008) have been successfully treated with ciprofloxacin. However, the outbreaks in Spain and Turkey were likely caused by the less virulent type B form of *Francisella tularensis* (Hepburn and Simpson, 2008). The efficacy of ciprofloxacin against the more virulent type A strains in humans is less well understood. Animal models of infection with the type A strain Schu S4 suggest that orally delivered ciprofloxacin is only effective against type A systemic infections if administered within 24 h of challenge (Piercy et al., 2005). Furthermore, studies using an animal model of pneumonic infection with a type A strain, the most fatal form of the disease, suggest that orally delivered ciprofloxacin offers poor protection (Steward et al., 2006).

Enhancing cellular delivery of antibiotics by encapsulation in liposomes has been suggested as a method to improve antibiotic efficacy against intracellular pathogens (Pinto-Alphandary et al., 2000). Macrophages which are infected by *F. tularensis* can take up liposomes containing antibiotics, enabling intracellular delivery and a close proximity between the drug and bacteria. In addition, the inhalation of encapsulated drugs, such as ciprofloxacin, enables high sustained concentrations of drugs to be achieved in the lungs. For the pneumonic form of tularemia, this approach has an obvious advantage and, if administered soon after infection, encapsulated antibiotics could reduce or prevent the spread of disease and reduce mortality. Without encapsulation, small drugs such as ciprofloxacin are rapidly cleared after administration into the lung (Wong et al., 1995, 2003). This review describes early studies evaluating the efficacy of a prototype liposomal ciprofloxacin against *F. tularensis* and further development of the novel antibiotic preparation.

## Preliminary studies

An initial liposomal ciprofloxacin preparation comprised negatively charged liposomes prepared from phosphatidylcholine: cholesterol: phosphatidyl serine (in a ratio of 7:3:1) with 45% of the ciprofloxacin encapsulated (Di Ninno et al., 1993). Even with this very early formulation, results were very promising. Efficacy was evaluated in a murine model of tularemia in which mice were infected with *F. tularensis* live vaccine strain (LVS). The *F. tularensis* LVS strain is relatively avirulent in humans (and hence has been used historically as a live vaccine for humans) yet causes a lethal infection in mice and therefore is commonly used as a surrogate for more virulent strains. In mice challenged intravenously with 10^3^ CFU of *F. tularensis* LVS, a single intravenous dose of liposomal ciprofloxacin (50 mg/kg) provided a high level of protection if administered within 48 h of the challenge. Conversely, a single intravenous dose of unencapsulated ciprofloxacin (50 mg/kg) offered no measurable protection even when administered 24 h post-challenge (Di Ninno et al., 1993).

**Figure 1:**
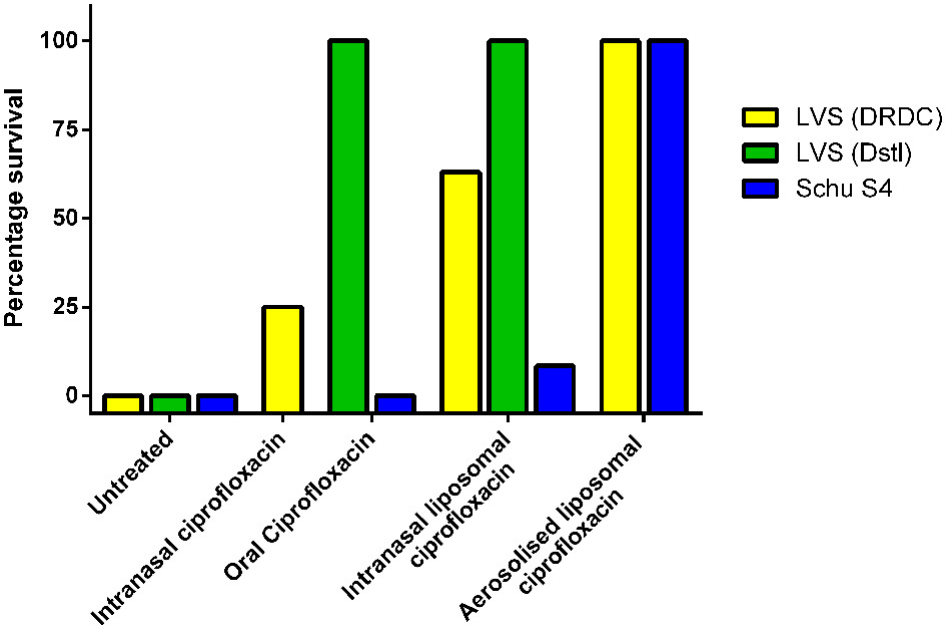
**Therapeutic efficacy of a single dose of ciprofloxacin or liposomal ciprofloxacin against murine inhalational *F. tularensis* LVS and Schu S4 infection**. Mice were challenged with *F. tularensis* LVS by the intranasal route (approximately 1 × 10^2^ CFU at DRDC or 6 × 10^4^ CFU at Dstl) or *F. tularensis* Schu S4 by the aerosol route (10 CFU retained dose). Treatment was initiated at 72 h post-challenge for LVS infections and 24 h post-challenge in the Schu S4 study. Treatment included 50 mg/kg of oral ciprofloxacin, 50 mg/kg of intranasal ciprofloxacin, 50 mg/kg liposomal ciprofloxacin, or 1 mg/kg lung dose of aerosolized liposomal ciprofloxacin. Graph shows percentage survival at the end of the experiment. LVS (DRDC) data is adapted from Di Ninno et al. (1993) (Intranasal ciprofloxacin and liposomal ciprofloxacin) and Wong et al. (2003) (aerosolized liposomal ciprofloxacin). Schu S4 and LVS (Dstl) data is adapted from Hamblin et al. (2014).

To model pneumonic tularemia, mice were challenged with 100 CFU of *F. tularensis* LVS via intranasal instillation. A single dose of intranasal liposomal ciprofloxacin (50 mg/kg) provided better protection than a single dose of intranasal ciprofloxacin (50 mg/kg) when therapy was initiated at 2 or 3 days post-challenge (Di Ninno et al., 1993; Wong et al., 1995) (Figure 1 shows 3 day data). The improved efficacy of intranasal liposomal ciprofloxacin compared to intranasal ciprofloxacin may be explained by the quick elimination of unencapsulated ciprofloxacin from the lung. Following intranasal instillation, unencapsulated ciprofloxacin is almost completely eliminated from the lungs within 2 h (Wong et al., 1995). Liposome encapsulation increased the half-life of ciprofloxacin in the mouse model from 1 to 10 h, and ciprofloxacin could still be detected in the lung of mice 24 h after dosing (Wong et al., 1995).

An alternative liposomal ciprofloxacin formulation, which encapsulated 90% of the ciprofloxacin, was developed using egg phosphatidylcholine and cholesterol in a 1:1 ratio (Conley et al., 1997). The pneumonic tularemia mouse model was used to evaluate the efficacy of this formulation delivered by the inhalational route. This delivery route is more representative, when compared to intranasal instillation, of the expected human pulmonary administration using a nebulizer or inhaler. In the pneumonic tularemia mouse model, a single dose of aerosolized ciprofloxacin (1 mg/kg lung dose) provided little or no protection whereas a single dose of aerosolized liposomal ciprofloxacin (1 mg/kg lung dose) offered 100% protection even when administered as late as 72 h post-challenge (Wong et al., 2003) (Figure 1 shows 72 h data). In addition, a single dose of aerosolized liposomal ciprofloxacin still offered a high level of protection (87%) when administered at 96 h post-challenge (Wong et al., 2003).

The efficacy of liposomal ciprofloxacin administered at such late time points after challenge is encouraging as late initiation of therapy increases the risk of a negative treatment outcome (Celebi et al., 2006). The use of liposomal ciprofloxacin may widen the window of opportunity for initiating therapy, providing more time for a *F. tularensis* deliberate release to be detected and for those affected to be successfully treated.

## Liposomal ciprofloxacin formulation development and human clinical trials

Liposomal ciprofloxacin has been further developed by Aradigm Corporation to improve encapsulation efficacy and increase the shelf life. More than 99% of the ciprofloxacin is encapsulated in this improved formulation, which has an extended shelf life of 24 months at 5°C (Cipolla et al., 2010). The human clinical development is summarized in Table 1. Aradigm Corporation initially conducted a Phase 1 study in healthy volunteers and Phase 2a studies in cystic fibrosis (CF) and non-cystic fibrosis bronchiectasis (BE) patients with the liposomal formulation CFI (Lipoquin®, also known as ARD-3100). The Phase 1 trials in healthy volunteers demonstrated the safety and tolerability of 300 mg CFI daily for 7 days (Bruinenberg et al., 2010). A Phase 2a study in patients with CF also demonstrated the potential to considerably increase lung ciprofloxacin concentrations by aerosol administration of CFI compared to orally administered ciprofloxacin. At steady state, a 500 mg oral dose of ciprofloxacin results in peak sputum concentrations of 1.86 μg/g in patients with CF (LeBel et al., 1986). In contrast, following administration of CFI the mean sputum concentration at day 7 was 88.4 μg/g (Bruinenberg et al., 2010).

**Table 1:** **Clinical phase 1 and 2 trials evaluating once daily dosing with CFI or DRCFI for treatment of patients with chronic *Pseudomonas aeruginosa* infection**.

**Purpose/indication**	**Formulation**	**Trial design and subjects**	**Main results**	**References**
Safety/tolerability/ pharmacokinetics	CFI	Phase 1, open-label, 20 healthy subjects. Treatment: doses of 150, 300, or 450 mg^a^ for 1 day; 300 mg^a^ for 7 days	Incidence of any adverse events was low; no serious or severe adverse events. Only three potential drug-related adverse-events, all mild, not dose-related	Bruinenberg et al., 2010
Safety/tolerability/ pharmacokinetics	CFI and DRCFI	Phase 1/2a, open-label, nine healthy and six BE^b^ subjects. Treatment: dose ranging for CFI and DRCFI	No clinically relevant reduction in FEV_1_^c^. All adverse events were mild (except for a urinary tract infection, not treatment-related)	Serisier, 2012
Cystic fibrosis	CFI	Phase 2a, open-label, single-arm, 22 well-treated adults with CF^d^. Treatment: 14 days with 300 mg^a^	Significant reduction in *P. aeruginosa* in sputum; 6.9% absolute increase in FEV_1_^c^ vs. baseline; exceptionally good pulmonary safety and tolerability compared to historical controls	Bruinenberg et al., 2010
Non-CF bronchiectasis	CFI	Phase 2a, open-label, single-arm, 36 BE^b^ adult patients. Treatment: 28 days of either 150 or 300 mg^a^	Significant reduction in *P. aeruginosa* in sputum; no difference between the two doses; no decline in lung function	Bruinenberg et al., 2010
Non-CF bronchiectasis	CFI	Phase 2b, randomized, double-blind, placebo-controlled, 95 adult BE^b^ patients. Treatment: 28 days of either 100 or 150 mg^a^ vs. matching placebos (ORBIT-1)	Both doses significantly reduced *P. aeruginosa* in sputum vs. placebo; treatment well tolerated—no need for pre-treatment or rescue with bronchodilators	Serisier, 2012
Non-CF bronchiectasis	DRCFI	Phase 2b, randomized, double-blind, placebo-controlled, 42 BE^b^ adult patients. Treatment: 150 mg^a^ vs. matching placebo for six cycles of 28 days on/28 days off (ORBIT-2)	Significant reduction in *P. aeruginosa* in sputum; median time to first pulmonary exacerbation more than doubled with DRCFI vs. placebo; DRCFI has better respiratory adverse effects profile than placebo	Serisier et al., 2013

a Expressed in terms of ciprofloxacin hydrochloride dose loaded in the nebulizer, of which approximately 15–20% is delivered to the lung.

b Bronchiectasis.

c Forced expired volume in 1 s.

d Cystic fibrosis.

The Phase 1 study with CFI also demonstrated the low systemic exposure of ciprofloxacin; following the 300 mg CFI dose, the average peak plasma concentration was only 0.1 μg/ml and an area under the curve (AUC) was 0.9 h·μg/ml (Bruinenberg et al., 2010). These values are considerably less than those for oral ciprofloxacin, where a 750 mg dose results in a peak concentration of 3.8 μg/ml and an AUC of 16.8 h·μg/ml (Lettieri et al., 1992). This reduction in systemic exposure may reduce ciprofloxacin associated side effects. It is believed that side effects or fear of side effects was responsible for 4% of US postal workers ceasing their ciprofloxacin medication during the 2001 USA anthrax attack (Brechner et al., 2001). Therefore, the use of inhaled CFI rather than oral ciprofloxacin may improve compliance with a prophylaxis regimen.

Prior to entry into Phase 2b studies, Aradigm Corporation evaluated an additional formulation, dual release ciprofloxacin for inhalation (DRCFI, Pulmaquin®, also known as ARD-3150) in a second Phase 1 study, with the view that a mixed pharmacokinetic profile combining the benefits of slow release with a transient initial peak of ciprofloxacin could provide incremental benefits for patients. This formulation was the result of mixing equal volumes of CFI and a solution of free ciprofloxacin. The DRCFI treatment was well tolerated in the Phase 1 study (Serisier, 2012).

Subsequently, both CFI and DRCFI have been investigated in patients with non-cystic fibrosis BE against placebo into two Phase 2b trials (ORBIT-1 and 2). In ORBIT-1, both doses of CFI were well tolerated and significantly reduced *P. aeruginosa* in sputum vs. placebo (Serisier, 2012). In ORBIT-2, DRCFI treatment also significantly reduced *P. aeruginosa* in sputum (Serisier et al., 2013). Notably, the median time to the first pulmonary exacerbation was more than two times longer in the patients treated with DRCFI vs. those treated with placebo. The DRCFI therapy was also well tolerated with the respiratory adverse effects profile of the patients treated with DRCFI being better than that for patients treated with placebo (Serisier et al., 2013).

## Further evaluation of liposomal ciprofloxacin against *F. tularensis*

Utilizing the advanced product, CFI (Lipoquin®), the efficacy of liposomal ciprofloxacin against *F. tularensis* has been further evaluated. As ciprofloxacin prophylaxis is generally given orally, inhaled CFI was compared to oral ciprofloxacin. In mice challenged intranasally with approximately 6 × 10^4^ CFU of *F. tularensis* LVS, the efficacy of oral ciprofloxacin (50 mg/kg) and intranasally instilled CFI (50 mg/kg) could not be distinguished as a single dose of either formulation offered full protection against a lethal challenge, even when therapy was delayed until 72 or 96 h post-challenge (Hamblin et al., 2014) (see Figure 1 for 72 h data). This clearly highlights the limitations of using reduced virulence *F. tularensis* strains for evaluating the efficacy of antibiotics against tularemia.

To discriminate between the two formulations, a mouse model of infection with the more virulent *F. tularensis* Schu S4 was used. In these studies, mice were challenged with *F. tularensis* Schu S4 via the aerosol route, with each mouse exposed to approximately 10 CFU. A single dose of aerosolized CFI (1 mg/kg lung dose) provided full protection against a lethal aerosol challenge. In contrast, a single dose, or 3 or 5 days of twice daily oral ciprofloxacin treatment (50 mg/kg) did not prevent mortality, with all mice succumbing to the infection (Figure 1 shows the single dose data). However, the 3 or 5 days course of twice daily oral ciprofloxacin treatment did increase the time to death of infected mice when compared to PBS treatment (Hamblin et al., 2014). Ciprofloxacin, administered by the oral route, does enter the lungs and maximal concentrations are similar to those achieved after an aerosol dose of CFI. However, the clearance rate of ciprofloxacin from the lung is 4000-fold higher than aerosolized CFI. Therefore, the superior efficacy of CFI, compared to ciprofloxacin, against aerosolized *F. tularensis* may be due to the persistence of CFI in the lungs (Hamblin et al., 2014).

Interestingly, aerosolized CFI was found to be more effective than intranasally instilled CFI, as a single dose of intranasally instilled CFI (50 mg/kg) did not prevent mortality (>10% of the mice survived) (see Figure 1). This may be due to different lung distribution following the two routes of administration, since aerosolized CFI is distributed more uniformly throughout the lung. The high level of protection offered by CFI against a highly virulent strain of *F. tularensis* is encouraging and suggests further in-depth studies are warranted.

## Future work

Further evaluation of the efficacy of liposomal ciprofloxacin using mouse and non-human primate models of tularemia could support an application for licensure by the FDA under the animal rule. Such studies could determine the maximum window of opportunity for initiating therapy and the shortest regimens that are effective. Dose ranging studies could inform device selection by determining if an effective dose can be administered using a small portable hand-held inhaler or if a higher dose requiring delivery by a nebulizer is needed. For example, the AERx® inhaler developed by Aradigm Corporation can deliver 50 μl in each metered dose. In comparison, nebulizers can deliver more drug (6 ml of DRCFI in Aradigm Corporation's clinical trials) but are larger and require a power source. These studies could also enable a comparison of the two liposomal ciprofloxacin formulations, CFI and DRCFI, to determine which is more effective and therefore more appropriate as a tularemia therapy.

In addition, studies to date have not investigated the efficacy of inhaled liposomal ciprofloxacin against systemic tularemia, which can develop from the pneumonic form. Co-treatment of liposomal ciprofloxacin with an orally or intravenously delivered antibiotic may warrant investigation as this therapy regimen could enable successful treatment of pneumonic infections that have spread systemically.

## Conclusion

CFI is a promising therapy for pneumonic tularemia, having the potential to shorten the current prophylactic regimen used in the event of a deliberate release of *F. tularensis*. CFI also offers potential for enhanced therapeutic outcomes for the treatment of naturally occurring tularemia caused by highly virulent strains of *F. tularensis.* Further study of liposomal ciprofloxacin is warranted to fully determine the utility of this formulation as a tularemia therapy.

## Author contributions

Karleigh A. Hamblin, Jonathan P. Wong, James D. Blanchard, and Helen S. Atkins drafted the manuscript. The final manuscript was approved by Karleigh A. Hamblin, Jonathan P. Wong, James D. Blanchard, and Helen S. Atkins.

### Conflict of interest statement

James D. Blanchard is an employee of Aradigm Corporation and has patents on DRCFI (Pulmaquin). The authors declare that the research was conducted in the absence of any commercial or financial relationships that could be construed as a potential conflict of interest.
